# A novel PCFT gene mutation (p.Cys66LeufsX99) causing hereditary folate malabsorption

**DOI:** 10.1016/j.ymgme.2009.11.004

**Published:** 2010-03

**Authors:** Esther Meyer, Manju A. Kurian, Shanaz Pasha, Richard C. Trembath, Trevor Cole, Eamonn R. Maher

**Affiliations:** a Department of Medical and Molecular Genetics, Institute of Biomedical Research, University of Birmingham, Birmingham B15 2TT, UK; b Department of Paediatric Neurology, Birmingham Children’s Hospital, Steelhouse Lane, Birmingham B4 6NH, UK; c Department of Medical and Molecular Genetics, King’s College London, School of Medicine, 8th Floor Tower Wing, Guy’s Hospital, Great Maze Pond, London SE1 9RT, UK; d West Midlands Regional Genetics Service, Birmingham Women’s Hospital, Metchley Park Road, Edgbaston, Birmingham B15 2TG, UK

**Keywords:** Hereditary folate malabsorption, *PCFT*, *SLC46A1*, Frameshift mutation, Anemia

## Abstract

Hereditary folate malabsorption (HFM) is a rare autosomal recessive disorder which is characterized by impaired intestinal folate malabsorption and impaired folate transport into the central nervous system. Mutations in the intestinal folate transporter *PCFT* have been reported previously in only 10 individuals with this disorder. The purpose of the current study was to describe the clinical phenotype and determine the molecular basis for this disorder in a family with four affected individuals. A consanguineous family of Pakistani origin with autosomal recessive HFM was ascertained and clinically phenotyped. After genetic linkage studies all coding exons of the *PCFT* gene were screened for mutations by direct sequencing.

The clinical phenotype of four affected patients is described. Direct sequencing of *PCFT* revealed a novel homozygous frameshift mutation (c.194dupG) at a mononucleotide repeat in exon 1 predicted to result in a truncated protein (p.Cys66LeufsX99). This report extends current knowledge on the phenotypic manifestations of HFM and the *PCFT* mutation spectrum.

## Introduction

Folates are a family of vitamins that contribute one-carbon groups for the synthesis of nucleic acids and for the methylation of DNA, histones, and other molecules [1]. Mammals are unable to synthesize folates so that these essential nutrients must be obtained from dietary sources [2]. Hence, intestinal folate absorption is a critical element in folate homeostasis. Recently, the mechanism of intestinal folate absorption was clarified with the cloning of the proton-coupled folate transporter (PCFT)^1^ which was shown to be mutated in the autosomal recessive disorder, hereditary folate malabsorption [3–5]. Since the initial reports, three additional kindreds have been described with this disorder in which the *PCFT* gene was mutated [6–8].

Infants with HFM usually present in the first few months of life with failure to thrive, pallor, and often diarrhea. This is associated with megaloblastic anemia and hypoimmunoglobulinemia often resulting in severe infections. In many cases, there are also neurological deficits such as developmental delay, mental retardation, and seizures. This is related to a defect in the transport of folates into central nervous system as manifested by very low levels of folate in the cerebrospinal fluid [5]. Unless diagnosed early, HFM can be fatal or cause permanent neurological damage. However, if treatment with parenteral folate or high doses of oral folate is started early in infancy, the signs and symptoms of this disease can resolve completely and children can develop normally.

Here we report on the clinical course of four siblings with a mild form of HFM and demonstrate a mutation in the *PCFT* gene that results in the complete loss of the PCFT protein.

## Methods

### Subjects

A consanguineous family of Pakistani origin with four children affected with HFM was ascertained and recruited for molecular genetic analysis. Ethnically matched laboratory control samples were analysed to evaluate the significance of novel sequence variants. Participants gave informed consent; the study was approved by South Birmingham Local Research Ethics Committee and was performed in accordance with the Declaration of Helsinki.

### DNA extraction

Genomic DNA was extracted from peripheral lymphocytes by standard techniques.

### Genetic linkage analysis

Prior to *PFCT* gene sequencing we confirmed linkage with two microsatellite markers, D17S2196 at 17.21 Mb and D17S1863 at 25.95 Mb that flank the *PCFT* gene (located at 23.75–23.76 Mb) on chromosome 17 (further details on request).

### Mutational analysis

Mutational analysis of *PCFT* was carried out by direct sequencing. The genomic DNA sequence of this gene was taken from Ensembl (http://www.ensembl.org/index.html) and primer pairs for the translated exons flanking exon–intron boundaries were designed using primer3 software (http://fokker.wi.mit.edu/primer3/input.htm). Amplification was performed according to standard protocols with BioMix™ Red (Bioline Ltd., London, UK). For amplification of difficult fragments such as GC rich sequences either 10% DMSO or GC rich solution of FastStart Taq DNA Polymerase (Roche Diagnostics Ltd., Burgess Hill, UK) was added to get PCR product. PCR conditions were an initial denaturation step at 95 °C for 5 min followed 35 cycles 30 s denaturation at 95 °C, 1 min annealing at 57–61.5 °C (depending on fragment) and 1 min extension at 72 °C with a final extension at 72 °C for 5 min. The PCR products purified with MicroCLEAN (Web Scientific, Crewe, UK) were directly sequenced by Big Dye Terminator Cycle Sequencing System (Applied Biosystem) and cleaned up using the EDTA method of precipitation. Sequencing reactions were run on an ABI PRISM 3730 DNA Analyzer (Applied Biosystem) and were analyzed using Chromas software (http://www.technelysium.com.au/chromas.html).

## Results

### Clinical findings

**Fig. 1:**
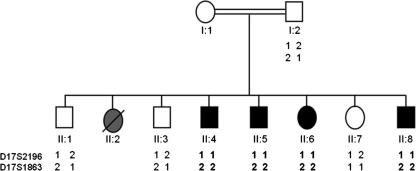
Pedigree and haplotype analysis of family with folate malabsorption. The analysis of two microsatellite markers of each side of the PCFT gene shows linkage to this locus as an identical homozygous haplotype is observed in all four affected individuals. Furthermore the unaffected siblings do not share the same genotype as the affected individuals.

Eight children were born to healthy consanguineous parents (first cousins) of Pakistani origin (Fig. 1). There was no family history of recurrent infection, haematological or neurological disorders. All children were born following a normal pregnancy and birth, and had an uneventful early neonatal course. Three children (II:1, II:3 and II:7) were healthy individuals with no evidence of haematological or neurological disease.

Child II:2 died at 3 months of age secondary to bronchopneumonia associated with severe anemia. On post-mortem examination, bone marrow histology revealed megaloblastic cellular changes. A provisional diagnosis of thalassaemia major was made (thalassaemia A carrier status was detected in both parents), although genetic confirmation of disease status was not undertaken.

Their fourth child (II:4), a male infant, presented at 3 months of age with pallor and feeding difficulties (weight 10th centile). General physical examination was normal and no dysmorphic features were evident. On haematological investigation he was found to be anemic (8.3 g/dl, normal range: 13.0–18.0 g/dl) and subsequent bone marrow examination confirmed a severe megaloblastic picture. Further biochemical investigations showed a markedly low folate level (serum folate: 0.58 ng/ml, normal range: 2.4–12.0 ng/ml; red cell folate: 104 ng/ml, normal range: 160–800 ng/ml), suggestive of a disorder of folate deficiency. Twice weekly injections of folinic acid (3 mg i.m.) were instigated and serum folate levels normalised within three weeks of starting therapy. At age three years, CSF investigation revealed a low folate (2.7 ng/ml, normal mean: 24 ng/ml). In early childhood, he made normal neurodevelopmental progress and there were no abnormalities on neurological examination. However from 8 years of age, there were concerns regarding some anti-social behavioural traits. At school, mild learning difficulties associated with a reduced attention span and problems with concentration were also reported. Formal cognitive assessment revealed that he was functioning at a level 2.5–3 years below the expected norm. At the age of 19 years he developed symptoms of myalgia and muscle fatigue. Nerve conduction studies and electromyelogram were normal.

The fifth child, (II:5) a male infant, also presented with feeding and breathing difficulties at age 2 months. His clinical presentation and disease course was very similar to his older brother.

In light of the family history, close monitoring of subsequent children was undertaken from birth. In patient II:6 and II:8, the red cell folate was normal at birth, and thus folinic acid treatment was initially deferred. However, both children presented in early infancy with vomiting, feeding difficulties and shortness of breath. In II:6, further clinical examination and cardiorespiratory investigation at presentation revealed a bronchopneumonia and fulminant heart failure.

Like their older brothers, both infants developed a megaloblastic anemia, and were also treated with folinic acid, following diagnosis of a low serum and red cell folate. Although there were concerns regarding cognitive and motor difficulties on developmental assessment of II:6 (age 4.5 years), these improved significantly during childhood. She proceeded to mainstream secondary school and there were no ongoing major concerns regarding school performance. Patient II:8 showed normal neurodevelopmental progress in childhood and adolescence. Unlike their older siblings, neither II:6 or II:8 had behavioural difficulties.

The patients are now age 25 (II:4), 23 (II:5), 19 (II:6) and 15 (II:8) years old. They all continue on twice weekly folinic acid treatment (3 mg i.m.) and remain asymptomatic (no major recurrent infections or neurological sequelae).

### Molecular genetic analysis

**Fig. 2:**
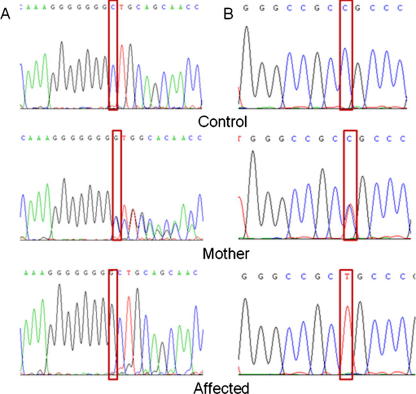
*PCFT* mutation analysis. (A) Duplication of a G in exon 1 (c.194dupG; p.Cys66LeufsX99). Top panel: sequence chromatogram of a control sample with wild-type allele, Middle panel: sequence chromatogram of the mother with the heterozygous *PCFT* mutation (c.194dupG). Bottom panel: sequence chromatogram of an affected individual with the homozygous *PCFT* mutation (c.194dupG). (B) C>T transition at nucleotide 340 in exon 2. Top panel: sequence chromatogram of a control sample with wild-type allele. Middle panel: sequence chromatogram of the mother with the heterozygous *PCFT* variant. Bottom panel: sequence chromatogram of an affected individual with the homozygous *PCFT* variant.

Molecular genetic investigation was undertaken using a positional-candidate gene approach. Thus genetic linkage studies with flanking markers (D17S2196 and D17S1863) were consistent with linkage to the *PCFT* gene (maximum LOD score of 2.68 at *θ* = 0.0 at D17S1863; Fig. 1) and direct sequencing of the *PCFT* gene was then undertaken. A novel homozygous frameshift mutation in exon 1 (c.194dupG, NM_080669.3; Fig. 2) that was predicted (in the absence of nonsense-mediated RNA decay) to result in a truncated protein (p.Cys66LeufsX99, NP_542400.2) lacking 296 amino acids from the C-terminus was detected in all affected individuals. The mutation was not detected in 364 ethnically matched control chromosomes and showed appropriate disease segregation within the family (the mother was heterozygous for the mutation and the unaffected siblings were either heterozygous or showed wild-type sequence). In addition a rare missense variant was detected that cosegregated with the frameshift mutation (c.340C>T; p.Arg114Cys). This variant was not detected in 564 control chromosomes.

## Discussion

The *PCFT* gene product has been implicated in folate receptor-mediated endocytosis by serving as a route of export of folates from acidified endosomes into the cytoplasm and regulates folate transport and absorption in intestine and other human tissues at low pH [3,9]. To date 9 different *PCFT* mutations (located in 4 of the 5 exons) have been reported in 10 ethnically varied kindreds. The novel frameshift mutation (c.194dupG; p.Cys66LeufsX99) we identified affects the same mononucleotide tract (consisting of 7 guanines) as a previously reported frameshift mutation that is predicted to result in a different truncated protein, (c.194delG; p.Gly65AlafsX25) [4]. This is consistent with the known hypermutability of such sequence tracts. Previously c.194delG was shown to be associated with complete lack of protein expression [4]. Although information on *PCFT* mutation spectrum is, as yet, restricted we note that in addition to the two mutations at the exon 1 mononucleotide repeat, two mutations at the same arginine residue have been reported (p.Arg113Ser and p.Arg113Cys) (Table 1) indicating that this residue plays an important role in folate transport function [4,6]. However, three previously reported missense mutations, p.Arg113Cys, p.Arg113Ser and p.Arg376Trp occur at hypermutable CpG dinucleotides [4,6]. All six missense mutations affected residues that were located within, or in the direct vicinity, of the predicted twelve transmembrane domains.

Classically HFM presents clinically in early infancy with anemia. However, there is considerable phenotypic variation between individuals with HFM [5]. Early clinical recognition of HFM is important because significant morbidity and mortality may be prevented with prompt diagnosis and treatment. The phenotype appears to be milder in individuals where the diagnosis is made at an early stage in infancy. However, in the absence of a positive family history, the diagnosis may often be delayed. Certainly in the family we describe, it is notable that the two younger children (in whom treatment was commenced early) had fewer neurological sequelae (currently they are aged 19 and 15 years) than their two older siblings. Molecular genetic testing can thus facilitate early diagnosis and treatment. In families with identified disease-causing mutations, prenatal or early antenatal testing could predict disease status (prior to the postnatal drop in folate or haemoglobin levels) and thus allow early folate therapy in affected cases.

To date no genotype–phenotype correlations are apparent but functional analysis of some missense mutations (e.g. p.Arg113Ser [4]) has shown severe effects on transporter function similar to the effect of truncating mutations. Nevertheless, interfamilial variations in phenotype between kindreds with similar loss of function *PCFT* mutations suggest that factors other than the precise nature of the *PCFT* mutation influence clinical phenotype. Hence genetic and/or environmental modifier effects may also be implicated. Interestingly, *Pcft*-deficient mice have greatly increased homocysteine levels (which are also influenced by diet and genetic variants in other genes) that could contribute to pathophysiological manifestations of HMF [10]. Increased recognition of HFM and the identification of further families and *PCFT* mutations will provide opportunities to define possible genotype–phenotype correlations and further explore the basis of the marked phenotypic variability that is characteristic of this condition.

## Figures and Tables

**Table 1:** Known mutations in *PCFT* in association with HFM.

Kindred (-patient)	Nucleotidechange	Amino acid change	Mutation type	Exon	Ref.
1–1/2	c.1082–1 G>A	p.Tyr362_Gly389 del	Splice site	Intron 2	[3,8]
2	c.194delG	p.Gly65AlafsX25	Frameshift	1	[4]
3	c.337 C>A	p.Arg113Ser	Missense	2	[4]
4	c.439 G>C	p.Gly147Arg	Missense	2	[4]
5	c.1274 C>G	p.Pro425Arg	Missense	4	[4]
6	c.954 C>G;	p.Ser318Arg;	Missense	2;	[4]
	c.1126 C>T	p.Arg376Trp	Missense	3	
7	c.337 C>T	p.Arg113Cys	Missense	2	[6]
8	c.197_198 GC>AA	p.Cys66X	Nonsense	1	[7]
9–1/4	c.194dupG	p.Cys66LeufsX99	Frameshift	1	Current study

Modified from Zhao et al. [4]; Ref. = References.

## Footnotes

1 *Abbreviations used:* PCFT, proton-coupled folate transporter; HFM, hereditary folate malabsorption; MIM, mendelian inheritance in man; CSF, cerebrospinal fluid; DMSO, dimethyl sulphoxide; PCR, polymerase chain reaction; EDTA, ethyldiamintetracitrate; i.m., intra muscular.
